# Evolved pollution tolerance in Gulf killifish (*Fundulus grandis*) from the Corpus Christi Inner Harbor, Texas, USA

**DOI:** 10.1007/s10646-026-03144-2

**Published:** 2026-09-01

**Authors:** Rachel B. Walkup, Cadance Swearingen, London R. Steele, Kendall E. Greer, Jiwan Kim, Cole W. Matson

**Affiliations:** Department of Environmental Science, Center for Reservoir and Aquatic Systems Research, Baylor University, Waco, TX USA

**Keywords:** Evolutionary toxicology, Adaptation, Resistance, Evolutionary rescue

## Abstract

The Corpus Christi Inner Harbor in Texas is a major center of industrial activity and contains high levels of persistent pollutants, including dioxins, furans, polycyclic aromatic hydrocarbons (PAHs), and polychlorinated biphenyls (PCBs). The Gulf killifish, *Fundulus grandis*, is an estuarine species inhabiting the Gulf of Mexico. *F. grandis* populations from the Houston Ship Channel (Galveston Bay, Texas) have been previously shown to be resistant to PCB-induced cardiac teratogenesis via a recalcitrant aryl hydrocarbon receptor (AHR) pathway. In this study, embryos from two *F. grandis* populations collected from the Corpus Christi Inner Harbor were exposed to varying doses of PCB-126 and evaluated for heart deformities at 144 h post fertilization. Both populations displayed significant resistance to PCB-induced cardiac teratogenesis compared to a reference population, representing the second documented cluster of adapted populations of *F. grandis*. Additionally, these populations exhibited lower basal cytochrome P4501A (CYP1A) activity (as measured via EROD assay) and reduced inducibility, indicating that a recalcitrant AHR pathway is at least partially responsible for the observed PCB resistance. Finally, we found that PCB-induced cardiac teratogenesis and CYP1A induction were highly correlated at the population level; however, individual CYP1A activity did not predict cardiac deformity. We propose a theoretical model in which inter-individual variation in CYP1A response explains this discrepancy and offers a conceptual framework for understanding individual variation within adapted populations. Collectively, these findings provide new insight into the mechanisms underlying adaptive responses to persistent pollutants and further establish *F. grandis* as a valuable model for evolutionary toxicology.

## Introduction

Anthropogenic pollution has significantly and rapidly altered the natural world, introducing novel selective pressures that drive wildlife to adapt. Evolutionary toxicology is a specialized branch of ecotoxicology concerned with how species adapt to anthropogenic pollutants and how these chemicals shape the genetic and phenotypic makeup of entire populations. Dioxins and dioxin-like compounds (DLCs), including polycyclic aromatic hydrocarbons (PAHs) and polychlorinated biphenyls (PCBs), cause developmental and reproductive defects at environmentally relevant concentrations (Wassenberg et al. 2002), thus providing a strong selective pressure on populations in heavily polluted areas.

Dioxins and DLCs have a common mode of action: induction of the aryl hydrocarbon pathway (Hahn 2002; Hahn et al. 2006). The aryl hydrocarbon receptor (AHR) is a transcription factor that is induced by xenobiotics such as PCBs or PAHs and activates genes within the AHR gene battery that are involved in xenobiotic detoxification, including cytochrome P4501A (CYP1A) which plays a critical role in Phase I metabolism (Gonzalez 1988; Gu et al. 2000; Ma 2001; Wirgin and Waldman 2004). The AHR pathway is highly conserved across taxa, but fish and some other non-mammalian species possess multiple copies of the AHR gene, while mammals only possess one (Clark et al. 2010; Di Giulio and Clark 2015; Hahn 2002; Powell et al. 2000). In teleost fish, AHR2 is more widely expressed and more functionally important in terms of responsiveness to AHR-inducing compounds (Powell et al. 2000). However, despite the AHR pathway’s role in xenobiotic detoxification, chronic activation of this pathway has been linked to cancer in mammals (Wang et al. 2020; Griffith and Frankel 2024) and can lead to severe developmental malformations in fish (Clark et al. 2010; Jayasundara et al. 2015). Furthermore, it has been demonstrated that AHR2 knockdown in fish protects against dioxin- and DLC-induced teratogenicity (Antkiewicz et al. 2006; Clark et al. 2010; Brown et al. 2015).

A recalcitrant AHR2 phenotype has been shown to be advantageous for organisms in environments polluted with AHR-inducing compounds such as PCBs or PAHs. An estuarine species commonly utilized in evolutionary toxicology that has exhibited evolved tolerance to the effects of DLCs is the Atlantic killifish (*Fundulus heteroclitus*). Localized populations of *F. heteroclitus* from North American estuaries near EPA-designated Superfund sites exhibit high levels of resistance to persistent pollutants and reduced activity of the AHR pathway (Whitehead et al. 2011; Di Giulio and Clark 2015), and genes in the AHR signaling pathway are recurrent targets of selection in independent adapted populations (Reid et al. 2016; Osterberg et al. 2018).

The distribution of *F. heteroclitus* is limited to the Atlantic Coast of North America, but its closely related sister species, the Gulf killifish (*Fundulus grandis*), is found in similarly polluted environments along the Gulf Coast (Gonzalez et al. 2009). It has been documented that *F. grandis* inhabiting the highly industrialized Houston Ship Channel (HSC) in Galveston Bay display a reduced inducibility of the AHR pathway as well as resistance to cardiovascular teratogenesis (Franco et al. 2022; Oziolor et al. 2014, 2019). Currently, 12 populations of *F. grandis* from Galveston Bay and the HSC have been characterized, with populations closest to the most heavily industrialized section of the upper HSC displaying increased resistance to PCB-induced cardiac teratogenesis. In a large percentage of resistant population individuals and a small percentage of individuals from intermediate populations, a 77-kb genomic deletion that spanned *AHR1a* and *AHR2a* was found. This deletion likely originated from a relatively recent hybridization event with *F*. *heteroclitus* that was mediated by accidental human transport (Oziolor et al. 2019). It was inferred that this AHR deletion provided a large fitness advantage for individuals that possessed it, indicating that regulation of the AHR pathway is likely an important mechanism of resistance in pollution-tolerant populations of *F. grandis* in the HSC.

While the HSC is one of the most heavily industrialized areas in the United States, many other industrialized estuarine areas exist throughout the Gulf Coast, including Corpus Christi Bay. The Port of Corpus Christi is the sixth largest port in the United States and numerous industrial and petrochemical plants along the Tule Lake Channel constitute a major source of organic contaminants within the Corpus Christi Inner Harbor (CCIH) (Ray et al. 1983). Sites along the CCIH have high concentrations of contaminants of concern, including mercury, lead, zinc, PCBs, and PAHs (Maruya et al. 1997; Ward & Armstrong, 1997). The levels of PCB contamination found within the CCIH are comparable to those seen in the most inland portions of the HSC (Oziolor et al. 2018) and it is likely that *F. grandis* from the CCIH have been subject to similar selective pressures to those observed in the HSC.

In this study, we investigated two populations from the CCIH to determine if *F. grandis* from this area are resistant to PCB126-induced cardiac teratogenesis. This model AHR agonist was chosen based on its previous use in killifish research and the high levels of resistance to this contaminant observed in *F. grandis* from the HSC (Oziolor et al. 2014). Additionally, we utilized an *in ovo* EROD assay as a proxy for CYP1A activity to determine if CCIH populations display a recalcitrant AHR pathway, the mechanism through which other *Fundulus* populations have acquired resistance to DLCs. Vince Bayou, a previously characterized adapted population from the HSC, and Smith Point, an established non-adapted reference population from Galveston Bay, were also evaluated for PCB126-induced cardiac teratogenesis and EROD CYP1A activity to provide a direct comparison to levels of PCB resistance in previously studied *F. grandis* populations.

Additionally, this study evaluated the correlation between PCB126-induced cardiac deformities and EROD CYP1A activity, at both the population and individual levels. It has previously been shown that these two factors are well-correlated at the population level (Wassenberg et al. 2002; Oziolor et al. 2019). We hypothesized that in adapted populations, lower CYP1A activity in individuals would be predictive of protection from PCB126-induced cardiac deformities.

## Methods

### Fish collection and care

**Fig. 1:**
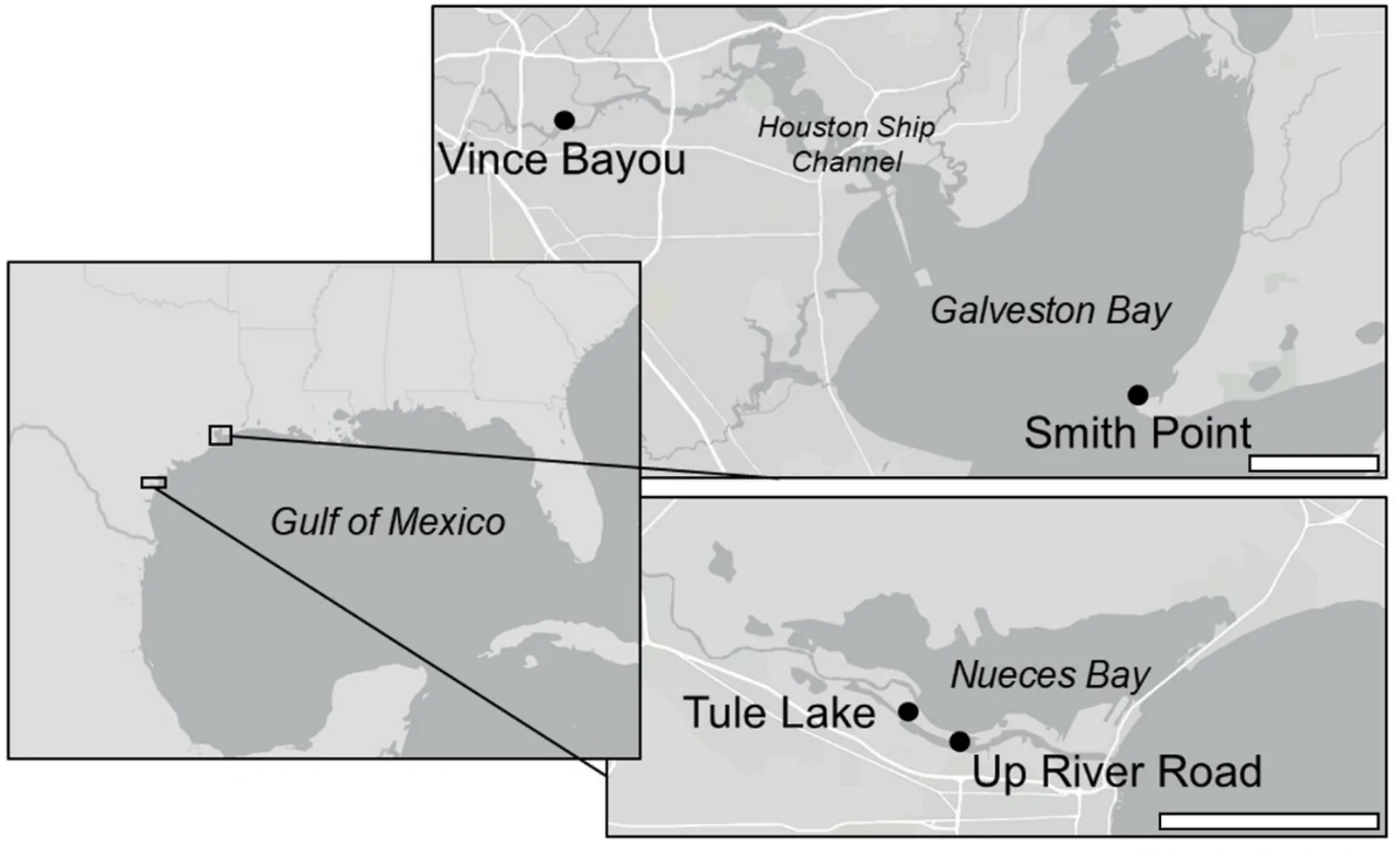
Collection sites for study populations of *F. grandis* from Galveston Bay, including the Houston Ship Channel, and the Corpus Christi Inner Harbor adjacent to Nueces Bay, on Corpus Christi Bay. Scale bar = 5 miles

Fish were collected from two contaminated sites in the Corpus Christi Inner Harbor (CCIH), Corpus Christi, Texas: Tule Lake (TL, 27°49’5.7” N; 97°30’7.5” W) and Up River Rd (UR, 27°48’52.5” N; 97°28’30.6” W) in April of 2022. Additionally, fish from two previously characterized populations from Galveston Bay, Texas were collected in late 2020 with Vince Bayou (VB, 29°43’10” N; 95°13’13” W) being a resistant population, and Smith Point (SP, 29°32’37” N; 94°47’08” W) serving as a reference population that does not demonstrate AHR recalcitrance or tolerance to DLCs (Fig. 1; Oziolor et al. 2019). These fish were maintained as P0 wild-caught adults in laboratory conditions until these experiments, and no multigenerational breeding occurred. Individuals in the present study retained phenotypes consistent with those previously reported for these populations (Oziolor et al. 2016, 2019).

After collection, fish were allowed to depurate for at least 1 month before starting experimental spawning. Fish were kept in 30 or 40-gallon glass tanks within a recirculating system with a 14:10 h light: dark cycle and water was maintained at ~ 10‰ salinity and ~ 25 °C. Twice daily, fish were fed pelleted food (PMI Nutritional International, LLC, Brentwood, MO, USA), flake food (Tetra Systems, Blacksburg, VA, USA), or frozen brine shrimp (San Francisco Bay Brand, Newark, CA, USA). All fish care and spawning practices were reviewed and approved by the Baylor University Institutional Animal Care and Use Committee.

During experimental runs, fish from each population were manually spawned once a week by pooling milt and oocytes. All individuals from a population sample were spawned during each experiment and experiments were only conducted if at least five females contributed eggs to the pool. After 1–2 h post fertilization (hpf), eggs were rinsed with 0.3% H_2_O_2_ to prevent bacterial and fungal infections (Ghomi et al. 2007) and rinsed three times with 10‰ artificial salt water (ASW). Rinsed eggs were then initially screened using a Nikon SMZ 1500 to ensure that all eggs were fertilized. At 24 hpf, eggs were again screened for initiation of gastrulation and normal development before being utilized in experiments.

### Chemical exposure

3,3’,4,4’,5-pentachlorobiphenyl (PCB126) was purchased from Absolute Standards (Hamden, CT, USA) and dimethyl sulfoxide (DMSO) was obtained from Fisher Scientific (Waltham, MA, USA). PCB126 stock solutions (10 and 1000 µg/mL in DMSO) were diluted with 10‰ ASW to generate nominal dosing concentrations of 0, 0.1, 0.5, 1, 10, 50, and 100 µg/L. 7-ethoxyresorufin (7-ER) was added in the PCB126 dosing solution at 24 hpf as part of the *in ovo* EROD assay from a 0.21 mg/L stock to a final concentration of 21 µg/L. DMSO concentrations were maintained at 0.02% throughout all doses and experimental runs.

After the 24 hpf embryo screening, five embryos were added to 50 mL of dosing solution in 100 mL hexane-rinsed glass jars. Embryos were incubated at 28 °C under a 14:10 h light: dark cycle until 144 hpf when *in ovo* heart deformity scoring and EROD assays were performed. All exposure treatments in a single experimental run were done in duplicate, and three independent experiments were conducted with the same embryos being used for both EROD assays and heart deformity scoring.

### Cardiac deformity scoring

At 144 hpf, the hearts of dosed embryos were assigned qualitative cardiac deformity scores. Deformities were assessed using a Nikon SMZ 1500 and cardiac deformity scores were assigned in a single blind manner. Scoring was performed on a three-point scale consisting of 0 (no defects and 2 heart chambers), 1 (moderate deformities and 1 heart chamber), and 2 (severely deformed and no distinct heart chambers) (Matson et al. 2008).

### Ethoxyresorufin-*O*-deethylase (EROD) assay

*In ovo* EROD assays were also performed at 144 hpf using methods previously described by Nacci et al. (1998) and modified by Wassenberg & Di Guilio (2004). Briefly, a solution of 7-ER at a concentration of 21 µg/L was included in all dosing solutions as described above. The dosed 7-ER is converted to fluorescent resorufin by CYP1A, which then accumulates in the bi-lobed urinary bladder of the embryos. Intensity of fluorescence in urinary bladders was measured at 144 hpf using a Nikon AZ100 with a Texas Red filter and 60x magnification with an exposure time of 60 ms.

A representative region of each urinary bladder was defined using a region of interest (ROI) stencil in ImageJ (ver1.53u) (Schindelin et al. 2012). A background measurement from a representative region outside of the urinary bladder was taken in each individual and the mean fluorescence value of this background was subtracted from the mean fluorescence value of the urinary bladder ROI. Individuals with a clearly damaged or malformed urinary bladder which failed to retain resorufin, a known problem in embryos with severe cardiovascular teratogenicity, were excluded from statistical analysis. If more than three individual embryos within a jar were severely damaged, the entire experimental unit was excluded and replaced. EROD activity was averaged within each jar and then normalized as a percentage of the Smith Point (reference) control group and/or population-specific controls for different analyses.

### Statistical analysis

All data analysis was performed using R and differences were considered to be significant at *p* ≤ 0.05. For each site and dose, embryos were exposed in replicate jars which were treated as the experimental unit, and dosing was repeated independently 3 times, yielding a total of six replicate jars per treatment (*N* = 6). For each replicate jar, all metrics collected from the five embryos within an individual jar were averaged prior to analysis to generate a single value for each endpoint. Data from both cardiac deformity scoring and EROD assays are presented as mean ± standard error. Between population comparisons for both deformity and EROD datasets were then performed using a two-way ANOVA to test the main effects of population and dose, as well as the population × dose interaction. This was followed by a Tukey’s post-hoc to evaluate pairwise differences. Homogeneity of variances was tested utilizing a Brown-Forsythe test. For individual level comparisons which did not fit ANOVA model assumptions, a non-parametric Kruskal-Wallis test was used for among-population comparisons.

Using cardiac deformities, 50% effective concentration (EC_50_) values were derived using a three-parameter log-linear model in the *drc* package in R (Ritz et al., 2015). Derived EC_50_ values from this study, together with previously reported EC_50_ values for Galveston Bay/HSC *F. grandis* populations (Oziolor et al. 2019), were linearly correlated with maximal CYP1A inducibility. Maximal CYP1A inducibility was normalized to a scale of 0-100 to account for differences in experimental design between the two studies.

Ordinal logistic regression was used to assess the association between EROD CYP1A activity and deformity severity (scored 0–2). Models included population and dose as covariates, and EROD × population interactions were tested using likelihood ratio tests.

## Results

### Cardiac teratogenesis

**Fig. 2:**
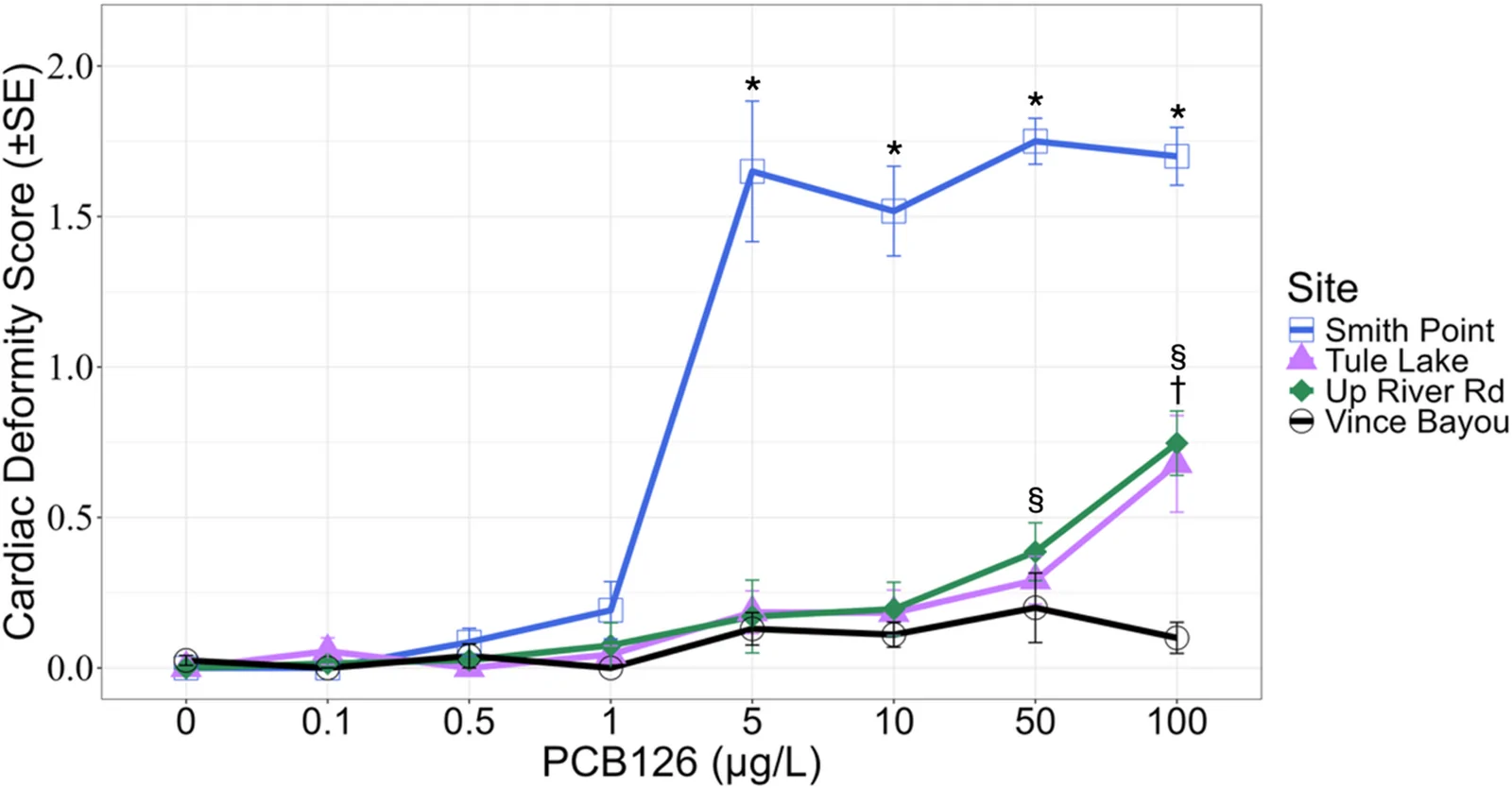
Developmental cardiac deformities in Corpus Christi Inner Harbor (CCIH) populations of *F. grandis* (Up River Rd and Tule Lake) as compared to the Vince Bayou HSC adapted population and a non-adapted reference (Smith Point) population from Galveston Bay. In a two-way ANOVA, the interaction term between PCB concentration and population was significant (*p* < 0.0001). The Smith Point reference population showed significant deformities starting at 5 µg/L (*p* < 0.0001) while the Vince Bayou adapted population did not show a significant increase in heart deformities at any of the tested doses. Conversely, CCIH populations did show significant increases in heart deformities at higher doses of PCB126. Symbols represent statistical significance compared to control for SP (*), TL (†), UR (§), and VB (‡)

From a two-way ANOVA, mean cardiac deformity scores significantly differed by site (*p* < 0.0001), PCB126 dose (*p* < 0.0001), and the interaction between these two factors (*p* < 0.0001) (Fig. 2). Significant cardiac deformities occurred in the Galveston Bay reference population (SP) starting at 5 µg/L relative to the corresponding within-population control (two-way ANOVA; Tukey’s post hoc: *p* < 0.0001). In contrast, it was found that the previously characterized adapted population from the HSC (VB) did not show significant deformities at any tested dose (two-way ANOVA; Tukey’s post hoc: *p* = 1.00). However, both CCIH populations (TL and UR) showed significant increases in cardiac deformities relative to their respective controls. These increases began at 50 µg/L for UR (two-way ANOVA; Tukey’s post hoc: *p* = 0.033) and at 100 µg/L in TL (*p* < 0.0001). Compared with the CCIH populations, VB differed significantly only at 100 µg/L (two-way ANOVA; Tukey’s post hoc: VB 100 vs. TL 100, *p* = 0.0006; VB 100 vs. UR 100, *p* < 0.0001).

### EROD CYP1A activity

**Fig. 3:**
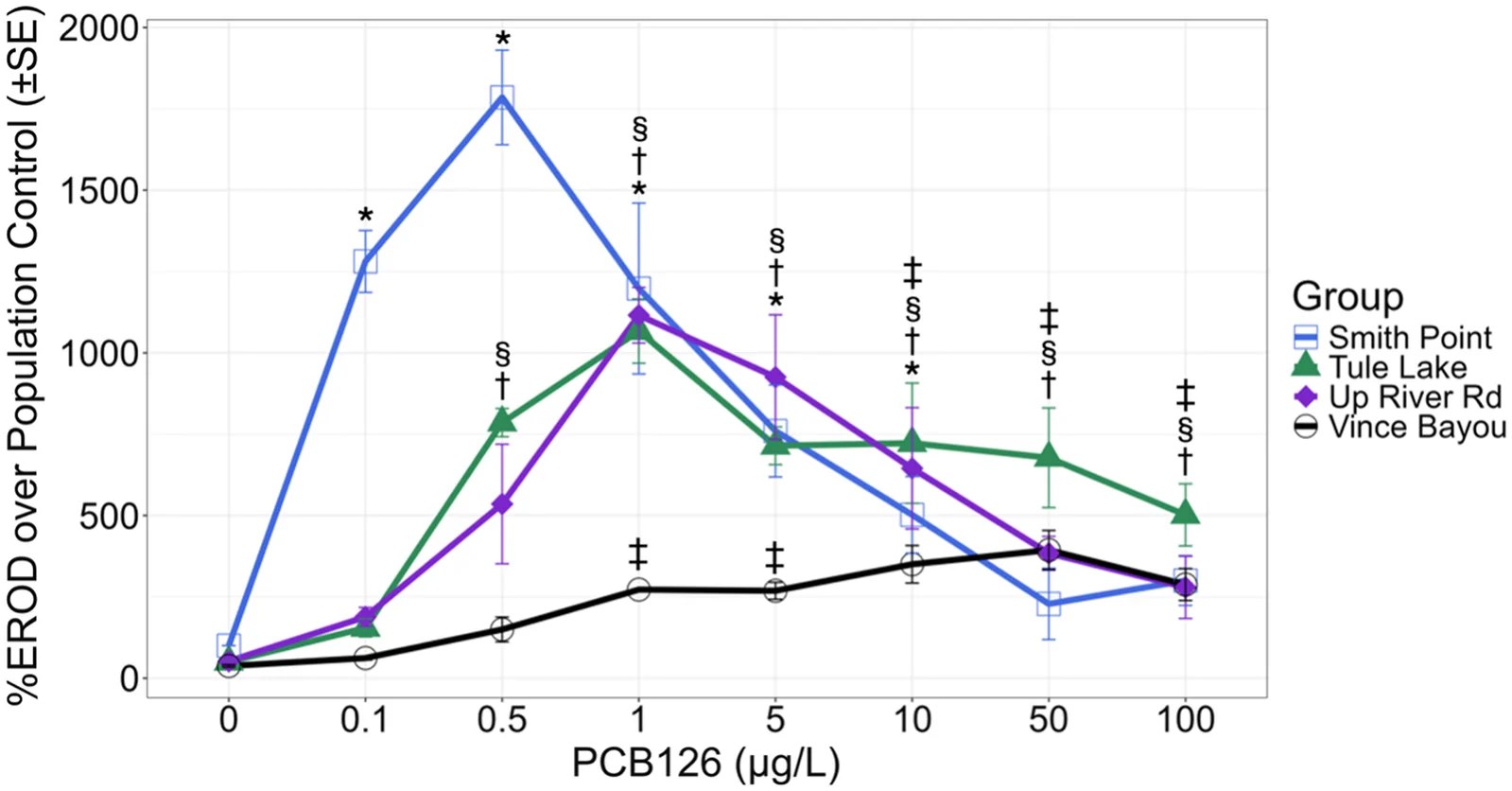
PCB126-induced EROD CYP1A activity in Corpus Christi Inner Harbor populations of *F. grandis* (Up River Rd [UR] and Tule Lake [TL]) with the adapted population (Vince Bayou [VB]) from the Houston Ship Channel and the reference population (Smith Point [SP]) from Galveston Bay. %EROD CYP1A over Population Control values were calculated by subtracting background fluorescence from urinary bladder ROI fluorescence, averaging values across all embryos in each experimental unit (jar), and normalizing each population to its vehicle control (defined as 100%). Two-way ANOVA showed that EROD CYP1A activity differed significantly by both population and PCB126 dose (*p* < 0.0001), and the interaction between these two factors was also significant (*p* < 0.0001). Overall, CYP1A induction significantly differed between SP and the two CCIH populations (two-way ANOVA followed by Tukey’s post hoc test; SP vs. UR, *p* = 0.0025; SP vs. TL, *p* = 0.0074). Symbols represent statistical significance compared to the corresponding control for SP (*), TL (†), UR (§), and VB (‡)

CYP1A activity was evaluated utilizing an *in ovo* EROD assay. EROD CYP1A values were calculated by subtracting background fluorescence from urinary bladder ROI fluorescence, and were then normalized to each population’s vehicle control which was defined as 100%. EROD CYP1A activity differed significantly among populations and PCB126 doses (two-way ANOVA; *p* < 0.0001) (Fig. 3). The CCIH populations differed significantly from SP (SP vs. UR, *p* = 0.0025; SP vs. TL, *p* = 0.0074) but did not differ significantly from each other when evaluated separately from HSC populations. Significant CYP1A induction in SP embryos began at the lowest dosage point (0.1 µg/L, *p* < 0.0001), whereas induction in the CCIH populations was not significant until 0.5 µg/L compared to within-population controls. CYP1A induction in both CCIH populations peaked at 1 µg/L, and their maximal CYP1A induction at this peak was lower than that seen at the peak induction of SP. CYP1A activity in VB was not significantly induced until 1 µg/L (*p* = 0.008) and reached its maximal induction at 50 µg/L.

**Fig. 4:**
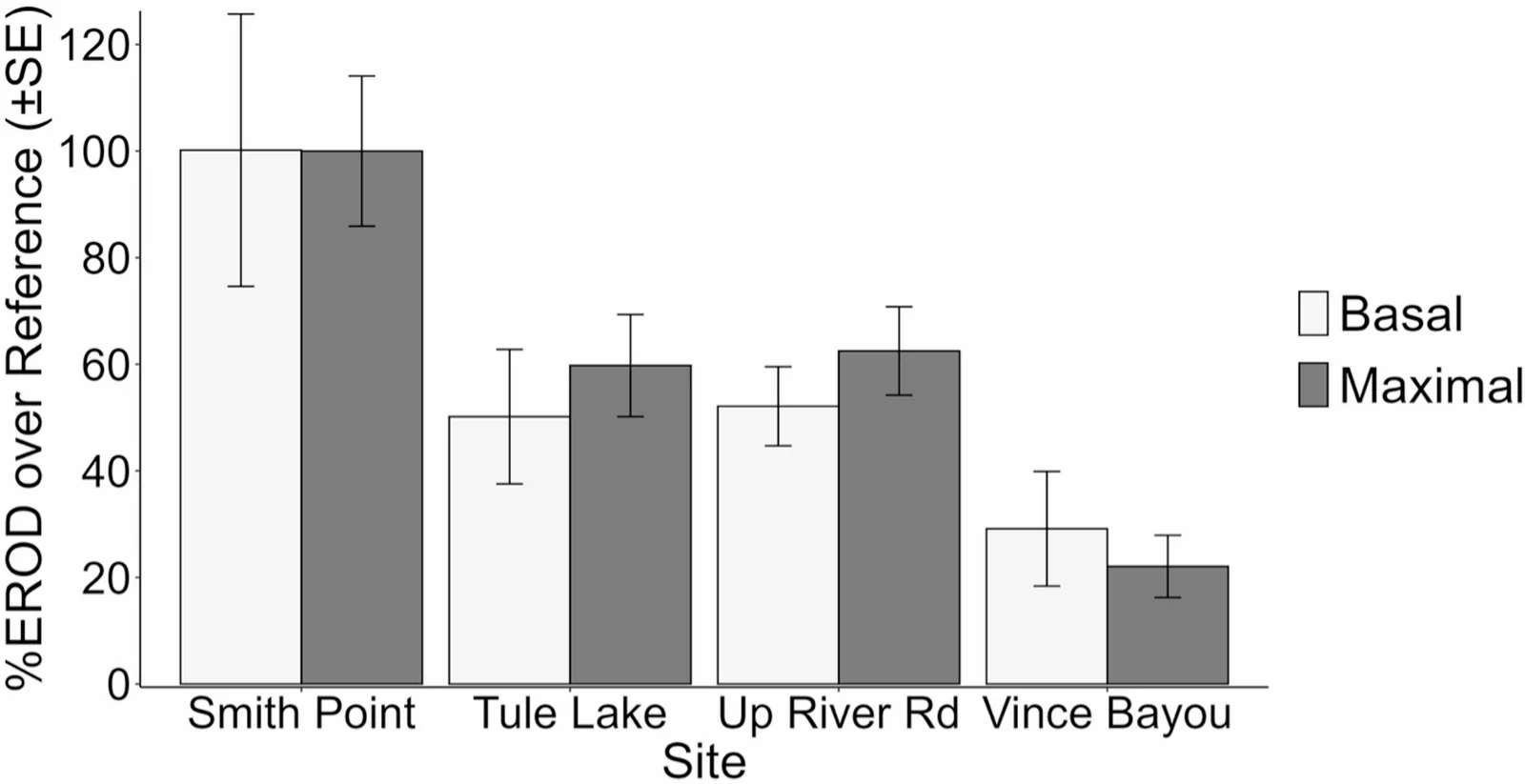
Basal and maximal EROD CYP1A activity in Corpus Christi Inner Harbor populations of *F. grandis* (Up River Rd [UR] and Tule Lake [TL]) with the adapted population (Vince Bayou [VB]) from the Houston Ship Channel and the reference population (Smith Point [SP]) from Galveston Bay. Relative EROD CYP1A activity was normalized to basal and maximal values measured in SP. Maximal CYP1A induction was defined as the highest observed response for each population, occurring at 0.5 µg/L for SP, 1 µg/L for UR and TL, and 50 µg/L for VB. Significant differences in basal CYP1A activity were observed only between SP and the other populations (one-way ANOVA followed by Tukey’s post hoc test; SP vs. TL, *p* = 0.019; SP vs. UR, *p* = 0.023; SP vs. VB, *p* = 0.0024). Basal and maximal CYP1A activity did not differ significantly within any population (two-way ANOVA followed by Tukey’s post hoc: TL, *p* = 0.988; UR, *p* = 0.973; VB, *p* = 0.997)

**Fig. 5:**
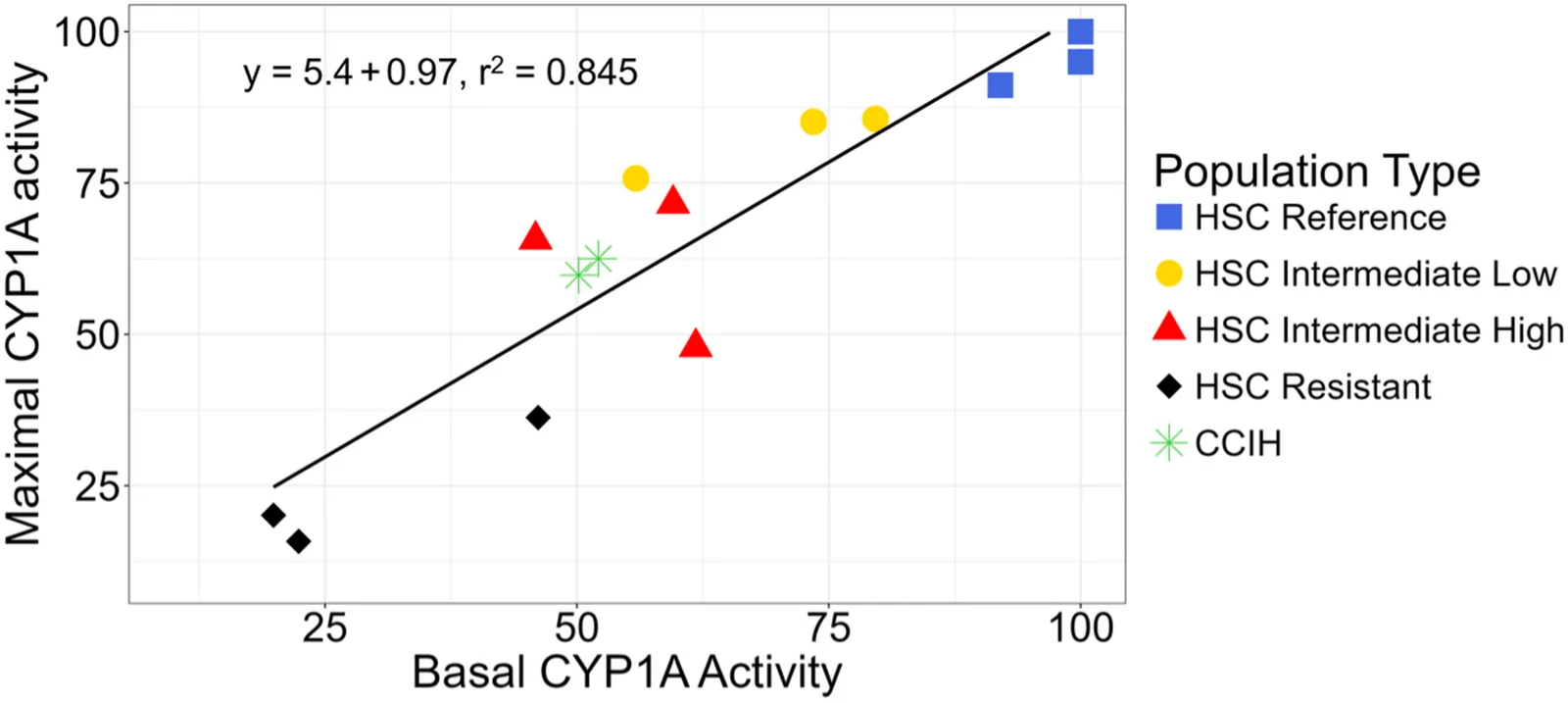
Correlation between basal and maximal EROD CYP1A activity in *F. grandis* populations from the Corpus Christi Inner Harbor (CCIH) and Galveston Bay/Houston Ship Channel (HSC). Basal and maximal EROD CYP1A activities were strongly linearly correlated across all evaluated populations (R^2^ = 0.845, *p* < 0.0001). Additionally, the regression line closely approximated a 1:1 relationship

Basal EROD CYP1A activity measured in vehicle controls, was significantly lower in TL, UR, and VB than in SP (one-way ANOVA; Tukey’s post hoc: SP vs. TL, *p* = 0.019; SP vs. UR, *p* = 0.023; SP vs. VB, *p* = 0.0024) (Fig. 4). Basal CYP1A activity did not significantly differ between the CCIH populations and VB (one-way ANOVA; Tukey’s post hoc: TL vs. VB, *p* = 0.41; VB vs. UR, *p* = 0.34). Maximal EROD CYP1A induction was defined as the highest observed response for each population and occurred at 0.5 µg/L for SP, 1 µg/L for UR and TL, and 50 µg/L for VB. Basal and maximal CYP1A activity were then normalized to the highest basal and maximal values observed in SP (100%). Within the adapted populations, no significant differences were observed between normalized basal and maximal CYP1A activity (two-way ANOVA; Tukey’s post hoc: TL, *p* = 0.988; UR, *p* = 0.973; VB, *p* = 0.997). This lack of significant differences indicates a reduced range of CYP1A activity in adapted populations relative to the reference population, consistent with attenuated AHR responsiveness. Furthermore, maximal and basal activity were found to be strongly linearly correlated across the CCIH populations and 12 previously characterized Galveston Bay/HSC populations, including SP (Reference) and VB (HSC Resistant) which were also included in this study (R^2^ = 0.845, *p* < 0.0001) (Fig. 5; Oziolor et al. 2019).

**Fig. 6:**
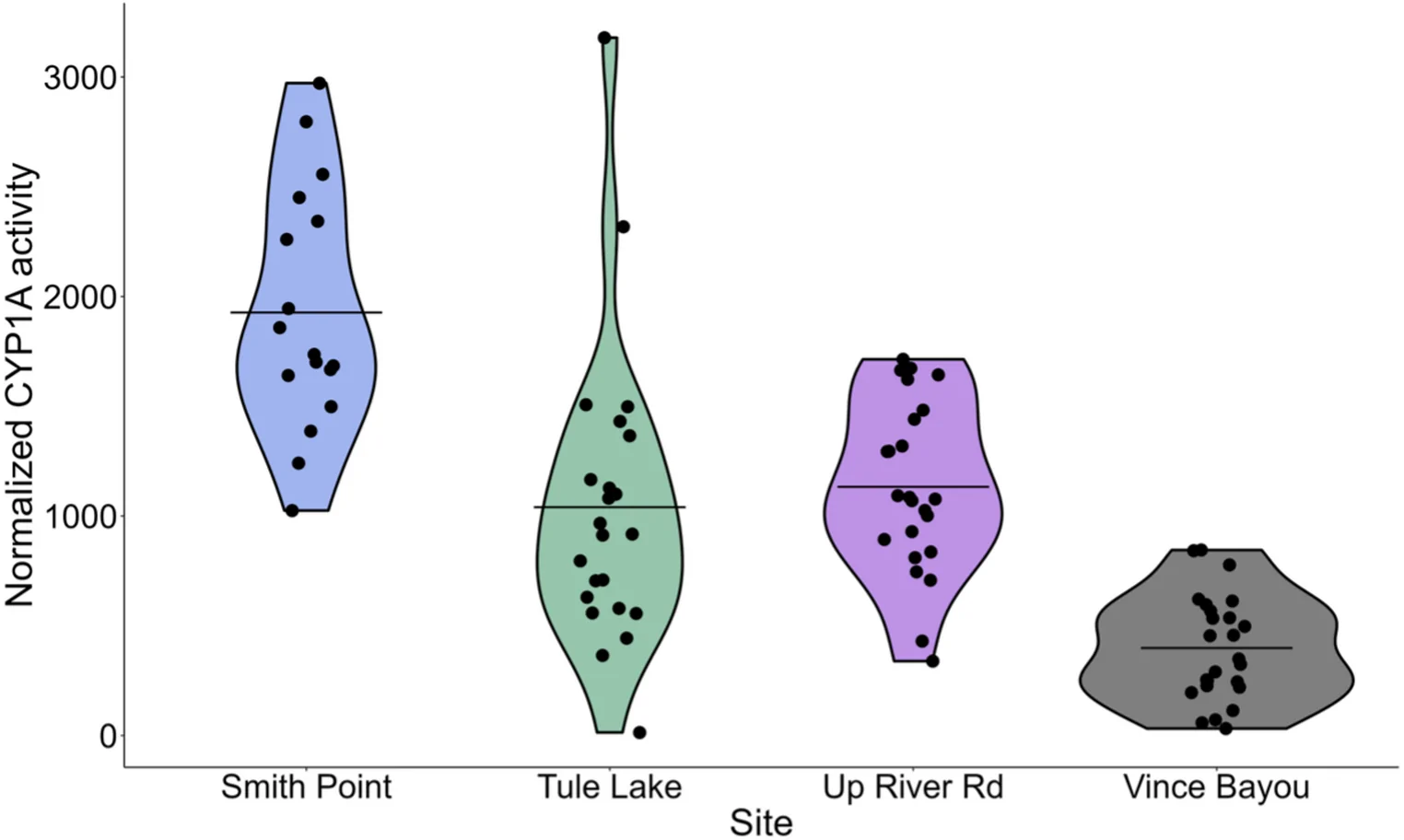
Comparison of individual maximal EROD CYP1A activity in *F. grandis* populations from Galveston Bay/Houston Ship Channel (HSC) (Smith Point [SP] and Vince Bayou [VB]) and the Corpus Christi Inner Harbor (Tule Lake [TL] and Up River Rd [UR]). Normalized EROD CYP1A activity was calculated by subtracting background fluorescence from urinary bladder ROI fluorescence and normalizing each individual value to mean population control values, with the mean control value defined as 100%. Maximal CYP1A induction was defined as the highest observed response for each population, occurring at 0.5 µg/L for SP, 1 µg/L for UR and TL, and 50 µg/L for VB. Individuals from the CCIH populations exhibited intermediate maximal CYP1A activity relative to SP and VB. VB showed lower inter-individual variability than the other populations, and heterogeneity of variance between populations was significantly different (Brown-Forsythe, *p* = 0.0044). TL and UR did not significantly differ in maximal CYP1A activity (Kruskal-Wallis, *p* = 0.99)

Maximal EROD CYP1A activity, varied not only among populations, but also greatly varied among individuals within the same population (Fig. 6). Values for maximal CYP1A induction in both CCIH populations overlapped with those observed in individuals from VB and SP. Individual CYP1A activity varied substantially in SP, UR, and TL while individual activity within VB was relatively more consistent. Consistent with this observation, a Brown-Forsythe test detected significant heterogeneity of variance in maximal EROD CYP1A inducibility among populations (*p* = 0.0044), suggesting that variation in CYP1A responsiveness differs across populations.

### EC_50_ values

**Fig. 7:**
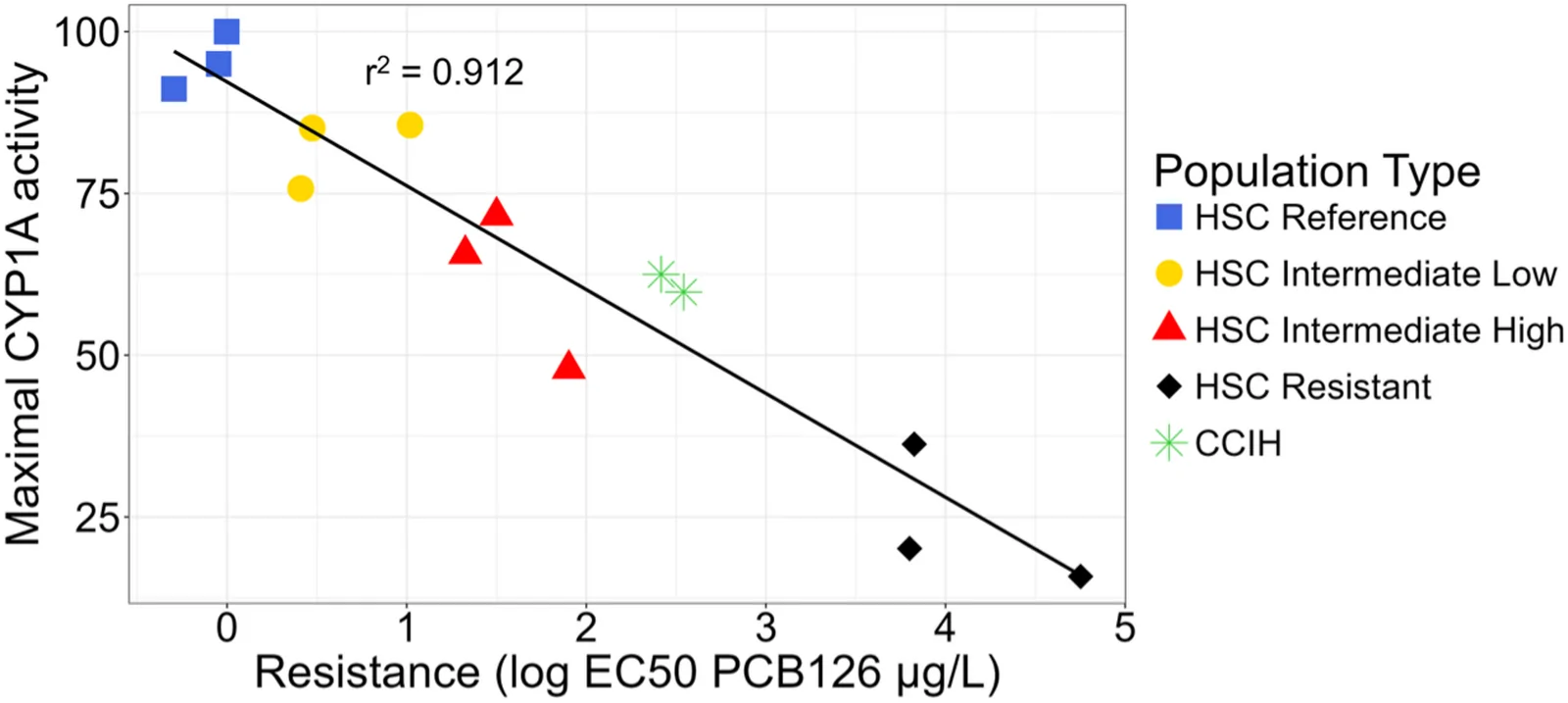
Correlation between EC_50_ and maximum EROD CYP1A activity in *F. grandis* from the Corpus Christi Inner Harbor (CCIH) and previously characterized populations from Galveston Bay/Houston Ship Channel (HSC). Across all populations, EC_50_ values and maximal EROD CYP1A activity were strongly correlated (R^2^ = 0.912, *p* < 0.0001). The CCIH populations had higher EC_50_ values compared to intermediate-high populations from the HSC but lower EC_50_ values when compared to populations classified as resistant

CCIH populations were substantially more resistant to PCB-induced heart deformities than the reference population (SP) (Fig. 7). After calculating EC_50_ values based on heart deformity scores, the EC_50_ values for CCIH populations were approximately 300-times higher than those calculated for the reference population. Across the CCIH and Galveston Bay/HSC populations, EC_50_ values were strongly correlated with maximal EROD CYP1A activity (R^2^ = 0.912, *p* < 0.0001). The CCIH populations lay between the HSC intermediate-high and resistant populations along the fitted regression.

### Relationship between heart deformities and EROD CYP1A activity at the individual level

**Fig. 8:**
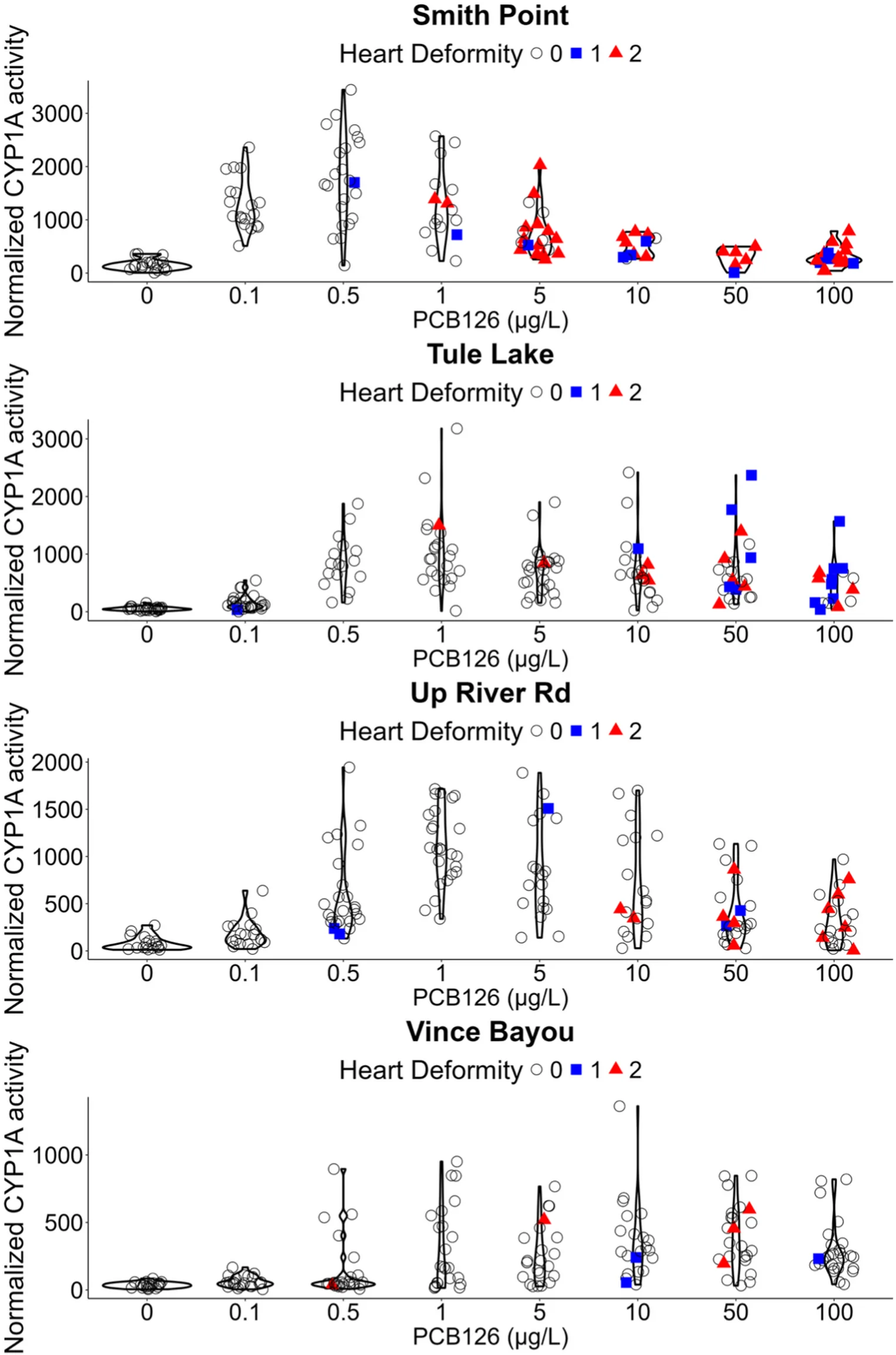
Distribution of individual EROD CYP1A activity and heart deformity scores in *F. grandis* populations from Galveston Bay/Houston Ship Channel (Smith Point and Vince Bayou) and the Corpus Christi Inner Harbor (Tule Lake and Up River Rd). Normalized EROD CYP1A activity was calculated by subtracting background fluorescence from urinary bladder ROI fluorescence and normalizing each individual value to mean population control values, with the mean control value defined as 100%. At the individual level, CYP1A activity was not significantly associated with heart deformities in the ordinal logistic regression models (β = 1.857 × 10⁻⁴, SE = SE = 2.182 × 10⁻⁴, *p* = 0.395), while both dose and population were significant predictors (*p* < 0.0001)

Individual EROD CYP1A activity, exhibited substantial variability at PCB126 doses that significantly induced CYP1A activity. As PCB126 dose increased, the incidence and severity of heart deformities also increased even though the dose at which these deformities began depended on the degree of resistance within the population (Fig. 8).

To formally evaluate the relationship between EROD CYP1A activity and deformity scores, deformity scores were analyzed using ordinal logistic regression. EROD CYP1A activity was not a significant predictor of deformity severity in the overall model (β = 7.271 × 10⁻⁵, SE = 1.824 × 10⁻⁴, *p* = 0.69) or the model including both population and dose as covariates in the model (β = 1.857 × 10⁻⁴, SE = 2.182 × 10⁻⁴, *p* = 0.395). Both population and dose were strong predictors of deformity severity in this model (*p* < 0.0001). Including the EROD × population interaction did slightly improve model fit (likelihood ratio test, *p* = 0.033); however, none of the individual interaction terms was significant (*p* > 0.6 across all populations), and analyses conducted separately for each population likewise showed no significant effects of EROD activity. Overall, EROD CYP1A was not a reliable predictor of deformity severity in individuals.

## Discussion

CCIH populations exhibited markedly greater resistance to PCB-induced cardiac deformities than our reference population (SP) (Fig. 2). This level of resistance was similar to that observed in HSC populations classified as having intermediate-high resistance (Oziolor et al. 2019). Resistance in the CCIH populations was lower than that observed in the highly resistant HSC population (VB), as embryos from VB showed no significant cardiac deformities across the tested PCB126 concentrations. Based on resistance mechanisms previously described in *F. grandis* (Oziolor et al. 2014, 2019), we evaluated CYP1A inducibility using an *in ovo* EROD assay in embryos exposed to PCB126. This was done to determine whether the CCIH populations possessed altered AHR pathway responsiveness that could confer resistance to PCB-induced cardiac deformities, similar to that reported in resistant populations of both *F. grandis* from the Galveston Bay/HSC and *F. heteroclitus* (Oziolor et al. 2014; Wassenberg et al. 2002). Compared with the SP population, the CCIH populations exhibited reduced maximal CYP1A inducibility, and peak induction occurred at higher PCB126 concentrations (Fig. 3). A similar pattern was also observed in the adapted HSC population (VB), although maximal induction occurred at a higher concentration than in the CCIH populations.

Resistant CCIH populations retained low levels of CYP1A inducibility following PCB126 exposure, suggesting that resistance is not mediated by complete loss of AHR pathway function, consistent with observations in other intermediately resistant populations from the HSC (Oziolor et al. 2019). The simultaneous reduction of both basal and maximal CYP1A activity suggests that the resistance mechanism suppresses overall AHR pathway responsiveness, resulting in proportional decreases in both maximal and basal CYP1A activity (Figs. 4 and 5). This reduction of maximal CYP1A activity was well correlated with protection from PCB-induced cardiac deformities at the population level. EC_50_ values for both resistant and sensitive populations were strongly correlated with maximal EROD CYP1A activity, consistent with previous data collected from populations along the pollution gradient in the HSC (Fig. 7). As this correlation remained strong when both CCIH and HSC populations were plotted together, this suggests that resistance to PCB-induced cardiac deformities is likely conferred by a similar mechanism in all of these populations, via downregulation of the AHR pathway.

While maximal EROD CYP1A activity was significantly reduced in CCIH populations, and it was strongly correlated with EC_50_ values for cardiac deformity at the population level, individual CYP1A activity varied greatly within these populations (Fig. 6), consistent with previous observations in intermediate-high populations from the HSC (Oziolor et al. 2019). Despite the strong association between PAH- and PCB-induced early-life stage cardiac deformities and CYP1A induction (Oziolor et al. 2019; Wassenberg et al. 2002), individual EROD CYP1A activity in the CCIH populations did not significantly predict heart deformity severity across PCB126 concentrations (Fig. 8). Although this result was contrary to our initial hypothesis, it provides further insight into the degree and pattern of adaptation in intermediate-high resistant populations and highlights additional factors that may contribute to individual variability.

**Fig. 9:**
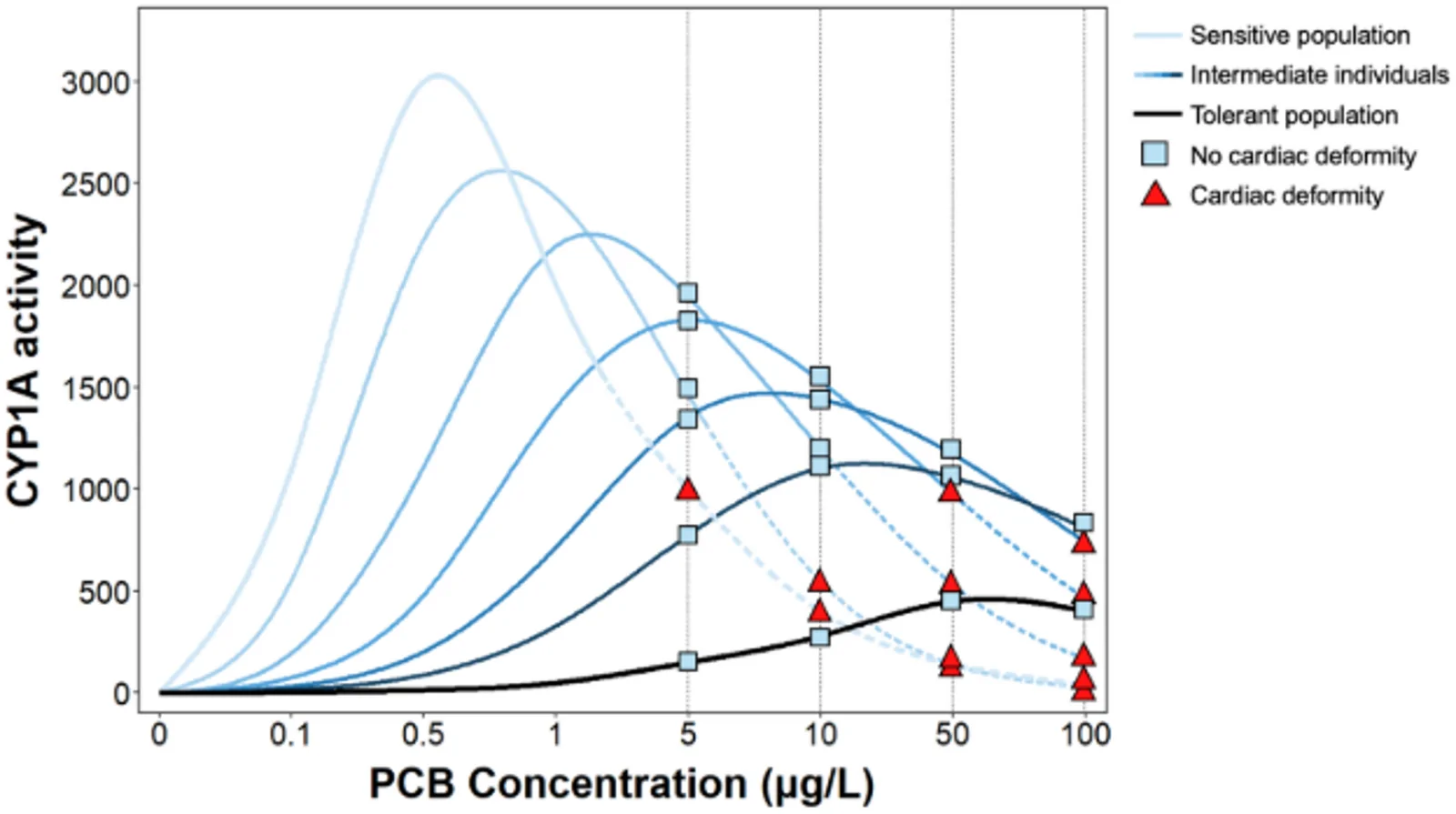
Theoretical model of inter-individual variation in CYP1A responses and cardiac deformity susceptibility. Light blue and black curves represent the dose-response profiles of sensitive and tolerant populations, respectively, while blue curves illustrate intermediate individual response profiles forming a continuum of CYP1A responses. Squares and triangles indicate the predicted absence and presence of cardiac deformity, respectively, at each PCB126 concentration. Solid line segments represent the predicted CYP1A response before the cardiac deformity threshold, whereas dashed line segments represent the response after the threshold has been exceeded. Cardiac deformity is hypothesized to occur once CYP1A activity declines to approximately 50% of its peak activity. This conceptual model illustrates how variation in individual CYP1A inducibility may result in different cardiac deformity thresholds among individuals

To reconcile the strong population-level association between CYP1A inducibility and cardiac deformity with the lack of individual-level correlation, we propose a theoretical model describing inter-individual variation in AHR responsiveness (Fig. 9). This model suggests that the onset of cardiac deformity occurs once CYP1A activity declines to approximately 50% of its peak activity on the right side of the dose-response curve, as supported by previous and current studies demonstrating that the reduction in CYP1A activity following maximal induction is associated with cardiac teratogenicity (Wills et al. 2010; Oziolor et al. 2014; Wassenberg and Di Giulio 2004; Matson et al. 2008). In this model, sensitive and tolerant populations represent the two extremes of CYP1A inducibility, while individual responses form a continuum of intermediate response curves. Consequently, individuals from the same population may reach the threshold for cardiac deformity at different PCB concentrations, even though they originate from the same polluted site. This provides a conceptual explanation for why maximal CYP1A activity poorly predicts embryo cardiac deformity at the individual level.

Based on the variability of maximal CYP1A induction observed in the TL and UR populations, these populations contain individuals with responses spanning those observed in the sensitive SP population to those observed in the resistant VB population (Fig. 6). Furthermore, greater inter-individual variation in CYP1A activity was observed in the CCIH populations at PCB concentrations exceeding those producing maximal induction, where cardiac deformities also began to occur and varied among individuals (Fig. 8). This variation was greater than that observed in the SP and VB populations, consistent with the proposed model in which individuals reach the threshold for cardiac deformity at different PCB concentrations. Therefore, such inter-individual variability may explain the lack of a significant association between EROD activity and cardiac deformity at the individual level across PCB concentrations. Essentially, at the population level CYP1A maximum inducibility is used, while at the individual level we only have CYP1A activity at one single PCB concentration, sometimes on the left side of the theoretical inducibility curve, sometimes on the right. We would still hypothesize that if one could estimate individual maximum CYP1A inducibility, that it would correlate with sensitivity to cardiac deformities.

However, we cannot rule out other possible sources of this inter-individual variation. CYP1A activity serves as a functional biomarker of AHR pathway activation but does not directly mediate cardiac teratogenicity, as CYP1A knockdown alone does not prevent pericardial edema while AHR knockdown does (Carney et al. 2004; Clark et al. 2010). Variation in EROD CYP1A activity may also reflect differences in contaminant uptake, biotransformation, oxidative stress responses, or the activity of other AHR targets, including other members of the CYP1 family that may also be detected by the EROD assay. Additionally, partial desensitization of the AHR signaling pathway in resistant individuals may further increase inter-individual variation by uncoupling receptor activation from downstream transcriptional responses.

*F. grandis* populations from the upper HSC gained at least some of their resistance through a hybridization event with *F. heteroclitus*, that was likely mediated by accidental human transport (Oziolor et al. 2019). CCIH and HSC populations are separated by approximately 200 miles, and *F. grandis* is an estuarine species that displays high site fidelity and limited gene flow between populations compared with species inhabiting more open marine environments (Williams et al. 2008). We have no evidence to suggest that the adaptive phenotype observed in CCIH populations evolved elsewhere, however, we can’t rule out this possibility. Future work will be needed to evaluate the evolutionary processes that gave rise to the observed resistance phenotype in CCIH populations. Despite this geographic isolation, resistance in CCIH populations was comparable to that observed in intermediate-high resistant populations from the HSC, where only a small fraction of individuals carry the introgressed AHR deletion. Because these intermediate-high HSC populations showed intermediate levels of resistance despite low frequencies of the AHR deletion haplotype (Oziolor et al. 2019), it is possible that multiple mechanisms, possibly including AHR-independent pathways, contribute to resistance in the CCIH populations. Population genomic and proteomic analyses would confirm whether resistance in the CCIH populations evolved independently or arose through shared ancestry or gene flow from adapted HSC populations, while also identifying additional mechanisms of pollution adaptation beyond the AHR pathway.

## Conclusions

This study identified the second known cluster of *F. grandis* populations resistant to persistent pollutants, demonstrating that populations from the Corpus Christi Inner Harbor exhibit intermediate-high resistance to PCB-induced cardiac deformities and reduced CYP1A inducibility. These findings support the hypothesis that similar pollution regimes can drive the evolution of comparable adaptive traits in geographically distinct estuarine populations. Furthermore, maximal CYP1A inducibility was strongly associated with resistance at the population level, indicating that reduced AHR responsiveness is a common characteristic of adapted killifish populations. The sister species of *F. grandis*, *F. heteroclitus*, is already a well-established model in evolutionary toxicology and multiple adapted populations have been identified in similarly polluted environments along the Atlantic Coast (Meyer et al. 2002; Nacci et al. 2002; Prince and Cooper 1995; Yuan et al. 2006). The discovery of a second cluster of adapted *F. grandis* populations offers a unique opportunity to better understand how pollutants along the Gulf Coast shape population-level adaptation and the genetic mechanisms through which species gain resistance to persistent pollutants.

Interestingly, CYP1A activity did not predict cardiac deformity at the individual level, despite the strong correlation between population-level CYP1A inducibility and cardiac deformity. To reconcile this discrepancy, we propose a theoretical model in which inter-individual variation in CYP1A response results in different sensitivity to embryo cardiac deformity among individuals, and that individual EROD activity measurements are not always predictive of individual inducibility. This conceptual framework provides new insight into how adaptive phenotypes may vary within resistant populations. Together, these findings further establish *F. grandis* as an important model for evolutionary toxicology and lay the foundation for future population genetic studies to elucidate the evolutionary origins and molecular mechanisms underlying adaptation to anthropogenic pollutants.

## Data Availability

Research data are available upon request to the corresponding author.
